# Geographical disparities and programmatic determinants of hydrocele surgery and lymphoedema management coverage for lymphatic filariasis in the Democratic Republic of the Congo, 2018–2024: A national analysis of routine programme data

**DOI:** 10.1371/journal.pntd.0014406

**Published:** 2026-06-02

**Authors:** Jean Claude Makenga Bof, Daniel Muteba

**Affiliations:** 1 National Neglected Tropical Diseases Programme, Ministry of Health, Kinshasa, Democratic Republic of the Congo; 2 Université Notre Dame du Kasayi, Kasai-Central, Kananga, Democratic Republic of the Congo; 3 School of Public Health, Université Libre de Bruxelles, Brussels, Belgium; University of Liverpool, UNITED KINGDOM OF GREAT BRITAIN AND NORTHERN IRELAND

## Abstract

**Background:**

Lymphatic filariasis (LF) is a significant neglected tropical disease in the Democratic Republic of the Congo (DRC). Although progress has been made toward interrupting transmission through mass drug administration, LF-related morbidities—particularly hydrocele, lymphoedema, and recurrent acute dermatolymphangioadenitis—continue to cause substantial disability, comorbid infections, and reduced quality of life. National evidence on the burden of LF morbidities and factors associated with access to morbidity management remains limited.

**Methods:**

We conducted a national retrospective analysis of routinely collected programmatic data from the National Neglected Tropical Diseases Control Programme in the DRC between 2018 and 2024. Data included estimates and management of LF-related hydrocele and lymphoedema across endemic provinces. Descriptive analyses assessed morbidity burden, geographic distribution, and temporal trends. Care cascade analyses were performed to quantify attrition across key stages of morbidity management. Multivariable linear regression models at provincial level were used to identify programmatic and health-system factors associated with hydrocele surgery coverage and lymphoedema management coverage.

**Results:**

A total of **8,471 hydrocele cases** and **5,310 lymphoedema cases** were identified nationwide. During the study period, **2,013 hydrocele surgeries (23.8%)** were performed, while 877 lymphoedema patients (16.5%) received the essential package of care. Marked geographic disparities were observed, with several high-burden provinces exhibiting particularly low coverage. Care cascade analyses revealed substantial attrition between case identification and receipt of care for both conditions. In multivariable analyses, hydrocele surgery coverage was positively associated with external partner support and availability of trained surgical personnel, while higher caseloads, post–Transmission Assessment Survey (post-TAS) surveillance phase, and geographic inaccessibility were associated with lower coverage. Lymphoedema management coverage was strongly associated with community-based care activities, health worker training, and availability of basic hygiene supplies.

**Conclusions:**

LF-related morbidities remain a substantial and unevenly addressed public health burden in the DRC, including in provinces that have achieved or are approaching interruption of transmission. Strengthening and scaling up morbidity management and disability prevention services—particularly for lymphoedema and hydrocele—are essential to improve patient quality of life and to meet World Health Organization requirements for elimination of lymphatic filariasis as a public health problem.

## Author summary

Lymphatic filariasis is a disabling neglected tropical disease that can cause swelling of the legs (lymphoedema) and scrotal swelling in men (hydrocele). These conditions lead to pain, recurrent infections, social stigma, and reduced ability to work. In the Democratic Republic of the Congo, thousands of people continue to suffer from these complications even though medicines are distributed every year to stop transmission of the infection. In this study, we analysed national data collected between 2018 and 2024 by community drug distributors during door-to-door drug distribution campaigns. These community workers identified people with possible lymphoedema or hydrocele and referred them to health facilities for care. We found large differences between regions in both the number of cases and access to treatment. Many patients were identified but did not receive surgery for hydrocele or care for lymphoedema. Access to treatment was better in areas where health workers were trained, where hygiene supplies were available, and where external partners provided support. Our findings highlight the need to strengthen care services at the health zone level, improve early detection by community workers, and expand access to simple diagnostic methods such as transillumination for hydrocele. Improving these services is essential to reduce suffering and achieve elimination of lymphatic filariasis as a public health problem.

## Introduction

Lymphatic filariasis (LF) is a debilitating neglected tropical disease (NTD), affecting impoverished populations in tropical and subtropical regions worldwide [1]. The disease is caused by mosquito-borne filarial parasites and is characterised by chronic clinical manifestations, including surgically treatable hydrocele, irreversible lymphoedema, and recurrent acute dermatolymphangioadenitis (ADLA) [2]. These conditions not only cause physical disability but also lead to profound social stigma, psychological distress, reduced productivity, and long-term socioeconomic hardship for affected individuals and their households [3].

To address the global burden of LF, the World Health Organization (WHO) established the Global Programme to Eliminate Lymphatic Filariasis (GPELF), built on two complementary pillars: interruption of transmission through mass drug administration (MDA), and morbidity management and disability prevention (MMDP) for individuals already affected by chronic manifestations [4]. While substantial progress has been made toward reducing transmission, the MMDP component has often received comparatively less investment and remains insufficiently implemented in many high-burden settings [5]. Ensuring availability of hydrocele surgery, hygiene-based lymphoedema care, management of ADLA episodes, and psychosocial support is essential for improving quality of life and achieving WHO validation of elimination as a public health problem [6,7].

Despite WHO recommendations that all endemic countries provide an essential package of care for LF-related morbidity management, access in practice remains limited, unevenly distributed, and dependent on external partner support and local health system capacity [8]. Barriers include shortages of trained personnel, weak referral pathways, geographic inaccessibility, and limited availability of supplies required for lymphoedema self-care. Community-based detection systems led by community drug distributors (CDDs), who identify suspected cases during door-to-door MDA campaigns, are unevenly implemented. Moreover, although hydrocele can be reliably confirmed through transillumination, its use remains inconsistent in many endemic districts.

The Democratic Republic of the Congo (DRC) bears one of the highest LF burdens in sub-Saharan Africa [9]. Despite significant expansion of MDA, and despite several health zones approaching or achieving transmission interruption, LF-related morbidities remain widespread across the country [10]. National estimates indicate tens of thousands of individuals living with hydrocele or lymphoedema, many of whom have never received appropriate care [11]. In the DRC, co-endemicity with other filarial infections, combined with vast geographic distances, insecurity, poor road networks, and limited surgical capacity, complicates morbidity attribution and poses major operational challenges for the delivery of MMDP services [12].

Yet, nationally representative evidence on morbidity burden, geographic disparities in care coverage, and programmatic determinants influencing access to services remains scarce. Available data are fragmented, based on small-scale assessments, or limited to isolated hydrocele surgery campaigns [13]. Comprehensive analyses integrating burden estimates, care cascade evaluation, and identification of determinants of coverage are urgently needed to inform national strategic planning and support WHO verification of LF elimination [14].

In this study, we present the first national retrospective analysis of LF morbidity management in the DRC between 2018 and 2024 using routinely collected programme data. Our objectives were to: (i) describe the national burden and geographic distribution of hydrocele and lymphoedema; (ii) assess coverage and attrition along the morbidity management care cascade; and (iii) identify programmatic and health system factors associated with access to hydrocele surgery and lymphoedema care at provincial level. By addressing these gaps, this study provides actionable evidence for strengthening morbidity management and disability prevention services across endemic health zones in the DRC and similar high-burden settings.

## Methods

### Ethical considerations

This study used anonymised, aggregated programme data collected as part of national LF surveillance and morbidity management. No identifiable personal data were accessed.

The protocol was reviewed and approved by the Ethics Committee of the School of Public Health, University of Kinshasa (Approval No. ESP/CE/938/2025), in accordance with national regulations and international standards [15].

In line with World Health Organization guidance, formal individual informed consent was not required for this secondary analysis of routine public health programme data [15].

As this study involved secondary analysis of anonymised and aggregated routine programme data, no individual written or verbal informed consent was required.

### Study design and setting

We conducted a national retrospective programmatic analysis of lymphatic filariasis (LF) morbidity management in the Democratic Republic of the Congo (DRC) covering the period from January 2018 to December 2024. The study analysed routinely collected data from the National Neglected Tropical Diseases Control Programme (NTD-CP), which implements World Health Organization (WHO)–recommended strategies for LF elimination, including morbidity management and disability prevention (MMDP) [16].

The primary operational unit for planning, implementation, and reporting was the **health zone**, supervised by provincial NTD coordinations responsible for oversight, supervision, and validation of morbidity data [16]. The analysis included all 26 provinces (provincial coordinations) and 264 health zones with reported LF morbidity data during the study period. These units represent the national operational coverage of the LF morbidity management programme in the Democratic Republic of the Congo.

### Data sources

Data were extracted from multiple routine programmatic sources maintained by the National Neglected Tropical Diseases Control Programme (NTD-CP), including morbidity registers, annual provincial reports, hydrocele surgery campaign reports, and monitoring and evaluation databases.

Morbidity data were primarily generated through household-level case detection conducted by trained community drug distributors (CDDs) during door-to-door mass drug administration campaigns, in accordance with national programme guidelines and WHO recommendations [17,18]. Prior to field deployment, CDDs received programme-specific training on the identification of suspected hydrocele and lymphoedema cases, including recognition of chronic lower-limb swelling and scrotal swelling suggestive of filarial hydrocele, based on standard case definitions. Suspected cases were subsequently validated through supervisory review at health zone level. This community-based surveillance approach relies on door-to-door registries rather than health-facility reporting, thereby providing a broader and potentially more exhaustive representation of the morbidity burden at population level, while acknowledging the possibility of under-detection compared with formal clinical examination.

The aggregated dataset used in this analysis included:

- identified hydrocele and lymphoedema cases
- eligibility and receipt of morbidity management
- geographic distribution across health zones and provinces
- availability of trained personnel for morbidity management
- documentation of external partner support

Together, these complementary sources provided comprehensive national coverage of lymphatic filariasis morbidity and service delivery activities for the period 2018–2024.

A detailed summary of annual morbidity management activities by province and year is provided in S2 Table.

### Study population and case definitions

The study population comprised all individuals identified by the national programme as having LF-related hydrocele or lymphoedema during the study period.

**Hydrocele cases** were defined as males presenting with scrotal swelling consistent with LF and eligible for surgery after clinical assessment. Eligibility for hydrocele surgery was determined through clinical examination, including transillumination where appropriate, confirmation of filarial hydrocele, assessment of operability, and exclusion of contraindications for surgery [19].

**Lymphoedema cases** were defined as individuals presenting with chronic swelling predominantly affecting the lower limbs (legs), consistent with lymphatic filariasis-related lymphoedema, irrespective of disease stage, and eligible for the essential package of care [20].

### Morbidity management interventions

Hydrocele management consisted of surgical treatment performed during routine hospital services or organised surgery campaigns, following WHO-recommended surgical techniques [21].

Lymphoedema management followed the WHO essential package of care, including hygiene-based self-care, wound care, treatment of acute dermatolymphangioadenitis (ADLA), education, and community follow-up [22].

Access to these services varied according to health zone capacity, trained personnel, partner support, and geographical accessibility.

### Outcome measures

Primary outcomes included:

1. **Hydrocele surgery coverage**: proportion of identified hydrocele cases receiving surgery.
2. **Lymphoedema management coverage**: proportion of identified lymphoedema cases receiving the essential package of care.

Secondary outcomes included **care cascade attrition**, defined as losses between case identification, evaluation, and treatment [23].

### Explanatory variables

Explanatory variables were selected based on WHO guidance and evidence from LF elimination programmes, including:

- external partner support
- availability of trained surgical personnel
- number of trained health workers
- availability of lymphoedema care supplies
- total caseload
- geographic accessibility
- provincial Transmission Assessment Survey (TAS) status [24,25]

These variables represent programmatic and health-system determinants of MMDP coverage.

### Statistical analysis

Descriptive analyses summarised morbidity burden, geographic distribution, and coverage of morbidity management. Care cascade analyses quantified attrition at successive stages of care.

Multivariable linear regression models were fitted at the provincial level to assess associations between explanatory variables and hydrocele surgery coverage and lymphoedema management coverage. The provincial-level analytical dataset used for the regression models is provided in S1 Table. Regression coefficients were expressed as absolute percentage-point changes. Statistical significance was set at p < 0.05. Analyses were conducted using Stata version 17.0 (StataCorp, College Station, TX, USA) [26].

## Results

### Burden and distribution of lymphatic filariasis–related morbidities

Between 2018 and 2024, a total of 13,781 lymphatic filariasis (LF) morbidity cases were identified nationwide in the Democratic Republic of the Congo, including 8,471 hydrocele cases (61.5%) and 5,310 lymphoedema cases (38.5%) (Table 1). Data were available from all 26 provinces and 264 health zones included in the national LF morbidity management reporting system.

**Table 1:** Characteristics of lymphatic filariasis morbidity cases included in the analysis, Democratic Republic of the Congo, 2018–2024.

Characteristic	Hydrocele cases	Lymphoedema cases	Total
Total cases identified, n (%)	8,471 (61.5)	5,310 (38.5)	13,781 (100)
Cases receiving morbidity management, n (%)	2,013 (23.8)	877 (16.5)	2,890 (21.0)
Cases awaiting care, n (%)	6,458 (76.2)	4,433 (83.5)	10,891 (79.0)
Health zones with reported cases, n	264	252	264†
Health zones providing care, n (%)	67 (25.4)	49 (19.4)	67 (25.4)†
Sex distribution: male, n (%)	8,471 (100)	3,392 (63.9)	11,863 (86.1)
Sex distribution: female, n (%)	—	1,918 (36.1)	1,918 (13.9)
Reported recurrent ADLA, n (%)	Not applicable	3,200 (60.3)	3,200 (23.2)‡
Secondary skin infections, n (%)	Not applicable	2,100 (39.5)	2,100 (15.2)‡

• ADLA: Acute dermatolymphangioadenitis.

• Hydrocele morbidity management refers exclusively to surgical treatment.

• Lymphoedema morbidity management includes hygiene-based self-care, wound care, management of acute inflammatory episodes, and community follow-up.

• Sex distribution reflects the clinical presentation of LF-related morbidity: hydrocele affects only men, whereas lymphoedema affects both men and women. In the present routine programme dataset, lymphoedema cases were reported in both sexes, with a higher proportion among men.

• Percentages in each morbidity category use the total identified cases for that morbidity as denominator; percentages in the total column use all identified LF morbidity cases (n = 13,781) as denominator.

• † Total health zones correspond to the maximum number reporting morbidity or providing care during the study period.

• ‡ Percentages in the lymphoedema column use n = 5,310; percentages in the total column use n = 13,781.

Hydrocele cases were reported in 264 health zones, while lymphoedema cases were reported in 252 health zones, confirming broad but heterogeneous geographic distribution.

Among all identified hydrocele cases, 2,013 patients (23.8% of all identified cases and 29.6% of clinically eligible cases) underwent surgery. Among identified lymphoedema cases, 877 patients (16.5%) received the essential package of care.

Although lymphoedema can affect both sexes, the routine programme data showed that reported cases occurred in both men and women, with a higher proportion among men (63.9%) than women (36.1%). This may reflect gender-related differences in community reporting, health-seeking behaviour, and possible under-detection of male cases during household-based case identification.

Overall morbidity burden and the distribution of hydrocele and lymphoedema by province and health zone are summarised in Table 1.

Year-by-year provincial summaries of morbidity management activities are presented in S2 Table.

### Provincial coverage of morbidity management

The analysis included all 26 provincial coordinations and 264 reporting health zones. Population denominators and reported case rates per 100,000 population covered are presented in Table 2. Coverage of morbidity management varied markedly across provinces (Table 2). Hydrocele surgery coverage ranged from 0% to 69.1%, with higher coverage in provinces receiving sustained partner support and demonstrating stronger surgical capacity. Several high-burden provinces, including Kasaï Central and Ituri, reported very low or no surgical coverage.

**Table 2:** Provincial coverage and reported case rates of lymphatic filariasis morbidity management services in the Democratic Republic of the Congo, 2018–2024.

Province/ Coordination	Population covered, n	Hydrocele cases, n	Hydrocele reported case rate/100,000 population	Hydrocele surgeries, n	Surgery coverage, %	Lymphoedema cases, n	Lymphoedema reported case rate/100,000 population	Cases receiving care, n	Care coverage, %
Kongo Central	231,718	77	**33.2**	49	63.6	118	**50.9**	21	17.8
Tshopo	3,397,835	758	**22.3**	275	36.3	1,203	**35.4**	138	11.5
Nord Ubangi	1,689,876	732	**43.3**	412	56.3	684	**40.5**	97	14.2
Kwilu	2,749,469	591	**21.5**	232	39.3	903	**32.8**	146	16.2
Kasaï Central	5,867,965	834	**14.2**	150	18.0	742	**12.6**	82	11.0
Kasaï	4,594,825	308	**6.7**	166	53.9	476	**10.4**	84	17.6
Maniema	2,944,450	284	**9.6**	112	39.4	355	**12.1**	63	17.7
Haut Katanga	398,736	68	**17.1**	47	69.1	126	**31.6**	27	21.4
Ituri	421,537	348	**82.6**	0	0.0	703	**166.8**	0	0.0
Other provinces*	9,352,835	4,471	**47.8**	570	12.7	1,000	**10.7**	219	21.9
**Total (national)**	**31,649,246**	**8,471**	**26.8**	**2,013**	**23.8**	**5,310**	**16.8**	**877**	**16.5**

• Hydrocele surgery coverage = (hydrocele surgeries performed ÷ identified hydrocele cases) × 100.

• Lymphoedema care coverage = (lymphoedema cases receiving care ÷ identified lymphoedema cases) × 100.

• Reported case rates per 100,000 population were calculated as (identified cases ÷ population covered) × 100,000.

• Population denominators correspond to the population covered by the reporting health zones and provincial coordinations included in the national LF morbidity management programme during the study period.

• Reported rates should therefore be interpreted as case rates among the covered population rather than province-wide prevalence estimates.

• Percentages and reported case rates are rounded to one decimal place.

• Provinces with 0% coverage indicate that no morbidity management activities were documented during the study period.

• *Other provinces include endemic provinces not listed individually and was aggregated to maintain consistency with national totals presented in Table 1.

**Fig 1:**
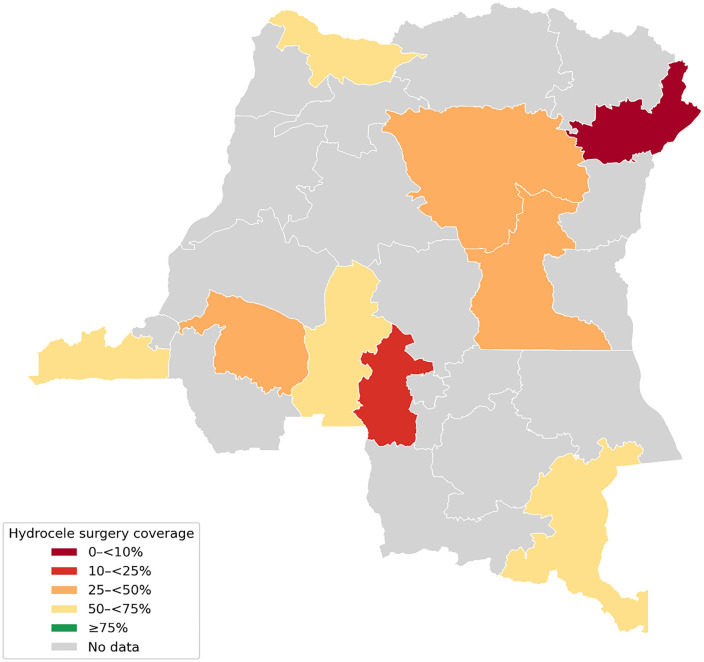
Provincial coverage of hydrocele surgery services in the Democratic Republic of the Congo, 2018–2024. Choropleth map showing the proportion of identified hydrocele cases that received surgical treatment across provinces between 2018 and 2024. Coverage was classified using programmatically relevant thresholds: 0– < 10%, 10– < 25%, 25– < 50%, 50– < 75%, and ≥75%. Provinces with no documented surgeries appear in the lowest category. The map highlights substantial geographic disparities in access to hydrocele surgery services, with several high-burden provinces reporting limited or absent coverage. All data were derived from routine programmatic reports compiled by community drug distributors and provincial NTD programme teams. Base map shapefile source: Natural Earth, public domain, compatible with CC BY 4.0. Grey provinces indicate non-endemic provinces or provinces with no reported morbidity data during the study period.

Lymphoedema management coverage was consistently lower than hydrocele surgery coverage, with several provinces reporting no documented lymphoedema care activities. Geographic disparities are illustrated in Fig 1.

Reported case rates among the covered population ranged from 6.7 to 82.6 hydrocele cases per 100,000 and from 10.4 to 166.8 lymphoedema cases per 100,000, indicating marked geographic heterogeneity in morbidity burden across provinces.

### Care cascade of morbidity management

**Fig 2:**
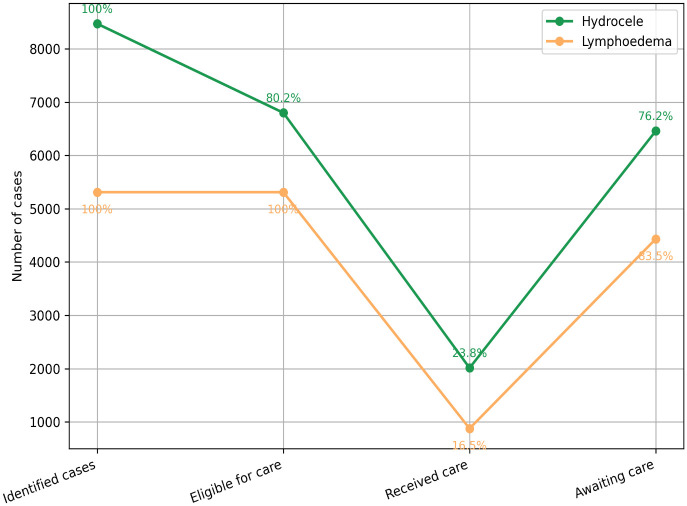
Care cascade of lymphatic filariasis morbidity management in the Democratic Republic of the Congo, 2018–2024. The figure illustrates the care cascade for lymphatic filariasis–related morbidities, showing the number and proportion of hydrocele and lymphoedema cases at successive stages of care. For hydrocele, the cascade distinguishes cases identified, cases clinically eligible for surgery, cases receiving surgical treatment, eligible cases still awaiting surgery, and cases deemed not eligible for surgery. Patients not eligible for hydrocele surgery are presented separately and are not included among those awaiting care. For lymphoedema, the cascade includes cases identified, cases receiving the essential package of care, and cases remaining without documented morbidity management by the end of the study period. Percentages are calculated using the total number of identified cases for each morbidity category as the denominator.

The care cascade analysis revealed substantial attrition between case identification, eligibility for care, and receipt of services (Table 3 and Fig 2).

**Table 3:** Care cascade of lymphatic filariasis morbidity management in the Democratic Republic of the Congo, 2018–2024.

Care cascade stage	Hydrocele cases, n (%)	Lymphoedema cases, n (%)	Total LF morbidity cases, n (%)
Cases identified	8,471 (100)	5,310 (100)	13,781 (100)
Cases clinically eligible for care	6,800 (80.2)	5,310 (100)	12,110 (87.9)
Cases receiving morbidity management	2,013 (23.8)	877 (16.5)	2,890 (21.0)
**Eligible cases awaiting care**	**4,787 (56.5)**	**4,433 (83.5)**	**9,220 (66.9)**
**Cases not eligible for hydrocele surgery**	**1,671 (19.7)**	—	**1,671 (12.1)**

**•** For hydrocele, patients deemed not eligible for surgery are presented separately and are not included among cases awaiting surgical management.

• All lymphoedema cases were considered eligible for morbidity management under WHO guidelines.

• Hydrocele morbidity management refers exclusively to surgical treatment.

• Lymphoedema morbidity management includes hygiene-based self-care, wound care, management of acute dermatolymphangioadenitis, patient education, and community-based follow-up.

• Percentages are calculated using the total number of identified cases for each morbidity category as denominator.

• Among all identified hydrocele cases, 76.2% did not receive surgery during the study period. However, only clinically eligible untreated patients (56.5% of all identified hydrocele cases) were classified as “eligible cases awaiting care.”

• “Eligible cases awaiting care” therefore represent clinically eligible patients who had not yet received morbidity management services by the end of the study period.

For hydrocele, 6,800 cases (80.2%) were clinically assessed and deemed eligible for surgery. Among these, 2,013 patients (29.6%) underwent surgical treatment, while 4,787 eligible patients remained awaiting surgery by the end of the study period. A further 1,671 identified hydrocele cases were deemed not eligible for surgery and were therefore analysed separately from those awaiting care. The higher proportion of hydrocele cases reported as awaiting care in Table 1 (76.2%) includes all patients who had not received surgery by the end of the study period, including those deemed not eligible for surgery, whereas the 56.5% reported in Table 3 refers specifically to clinically eligible patients still awaiting surgical management.

For lymphoedema, all 5,310 identified cases were considered eligible for care, of whom 877 (16.5%) received the essential package of care and 4,433 (83.5%) remained without documented morbidity management by the end of the study period.

### Factors associated with hydrocele surgery coverage

**Fig 3:**
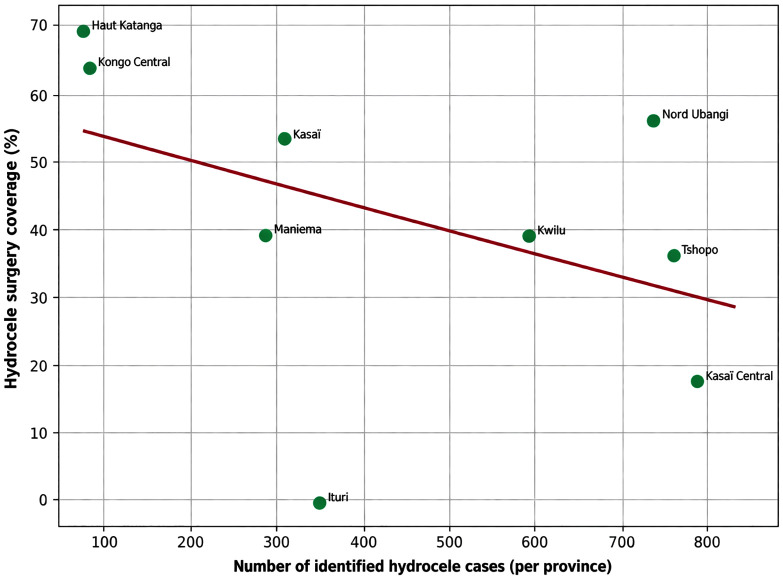
Association between hydrocele morbidity burden and surgery coverage in the Democratic Republic of the Congo, 2018–2024. Scatter plot showing the association between the number of identified hydrocele cases and hydrocele surgery coverage at the provincial level. Each point represents a province. The solid line represents the fitted linear regression, indicating an inverse relationship between morbidity burden and treatment coverage. Provinces with larger hydrocele caseloads tended to have lower surgical coverage, reflecting capacity constraints and geographic access challenges. Data are from aggregated routine programme sources.

Multivariable regression analysis identified external partner support, number of trained surgeons, and availability of operating room nurses as strong predictors of higher surgical coverage (Table 4). Conversely, high caseloads, post-TAS surveillance phase, and geographic inaccessibility were associated with significantly reduced coverage. These relationships are illustrated in Fig 3. The full provincial-level dataset used for these analyses is presented in S1 Table.

**Table 4:** Factors associated with provincial coverage of hydrocele surgery and lymphoedema management in the Democratic Republic of the Congo, 2018–2024.

Factors examined	Hydrocele – β coefficient (*95% CI*)	p-value	Lymphoedema – β coefficient (*95% CI*)	p-value
Community-based lymphoedema care activities	—	—	+21.4 (10.3 to 32.5)	<0.001
Training of health workers in lymphoedema care	—	—	+15.8 (5.9 to 25.7)	0.002
Availability of basic hygiene and washing kits	—	—	+12.6 (3.8 to 21.5)	0.006
External partner support	+18.6 (8.4 to 28.9)	0.002	+17.1 (6.4 to 27.8)	0.003
Number of trained surgeons (per additional surgeon)	+3.2 (1.1 to 5.4)	0.006	—	—
Number of trained operating room nurses (per 5 nurses)	+4.7 (1.9 to 7.5)	0.001	—	—
Total morbidity caseload (per 100 cases)	–2.1 (–3.8 to –0.4)	0.017	–3.4 (–5.6 to –1.1)	0.004
Post–Transmission Assessment Survey (post-TAS) phase	–11.4 (–20.6 to –2.3)	0.015	–9.2 (–17.6 to –0.9)	0.031
Geographical inaccessibility (hard-to-reach settings)	–9.8 (–18.1 to –1.6)	0.021	–11.7 (–21.9 to –1.6)	0.024
**Model constant**	22.5 (10.2 to 34.7)	<0.001	18.9 (7.1 to 30.7)	0.002

**Model summary**

*Hydrocele model*

• **Number of provinces analysed (N):** 26

• **R²:** 0.62

• **Adjusted R²:** 0.58

• **Overall model significance (F-test):** p < 0.001

*Lymphoedema model*

• **Number of provinces analysed (N):** 26

• **R²:** 0.66

• **Adjusted R²:** 0.63

• **Overall model significance (F-test):** p < 0.001

**Table Notes**

• β coefficients represent percentage-point changes in coverage associated with each factor.

• Positive values indicate increased coverage; negative values indicate reduced coverage.

• “—” indicates that the variable is not applicable for that morbidity category (e.g., surgical personnel not relevant to lymphoedema care).

• Variables were selected based on WHO programmatic guidance and operational relevance.

• All analyses were conducted at the provincial level using multivariable linear regression models.

• Caseload was scaled per 100 cases to improve interpretability of regression coefficients.

• Statistical significance was defined as **p < 0.05**.

### Factors associated with lymphoedema morbidity management coverage

Community-based lymphoedema care activities, health-worker training in the essential package of care, and availability of washing kits were significantly associated with higher coverage (Table 4). In contrast, higher caseloads, post-TAS phase, and hard-to-reach settings were associated with lower coverage.

### Summary of key findings

LF-related morbidities remain widespread and largely unmet in the DRC. Only one quarter of hydrocele cases and one sixth of lymphoedema cases accessed morbidity management. Significant geographic disparities persist, and attrition along the care cascade is substantial, particularly for lymphoedema. Programmatic capacity, partner support, trained personnel, and community-based delivery mechanisms were key determinants of coverage.

## Discussion

This national retrospective analysis provides the most comprehensive evaluation to date of the burden, coverage, and determinants of lymphatic filariasis (LF) morbidity management in the Democratic Republic of the Congo (DRC). Despite substantial progress toward interrupting LF transmission through large-scale mass drug administration (MDA), our findings clearly show that LF-related morbidities persist widely and remain largely unmanaged. Fewer than one-quarter of identified hydrocele cases and less than one-fifth of lymphoedema cases received appropriate care during the study period, underscoring critical weaknesses in the implementation of the morbidity management and disability prevention (MMDP) pillar of the elimination strategy.

### Persistent morbidity burden and programmatic gaps

The large number of hydrocele and lymphoedema cases identified between 2018 and 2024 confirms that morbidity persists long after transmission has declined, consistent with evidence from several endemic countries where morbidity remains substantial despite successful MDA programmes [27,28]. These findings highlight that interruption of transmission alone is insufficient to achieve WHO validation of LF elimination and that long-term investment in MMDP services remains essential.

Marked geographic disparities in morbidity burden and coverage further indicate profound inequities in access to care. Provinces with the highest burden were often those with the lowest coverage, reflecting a mismatch between needs and service availability. This inverse relationship between burden and coverage has been documented in other high-burden settings [29] and is exacerbated in the DRC by vast geographic distances, limited road networks, health workforce shortages, and logistical constraints.

### Differential coverage of hydrocele and lymphoedema management

Hydrocele surgery coverage was higher than lymphoedema management coverage across provinces, although still insufficient overall. This reflects the episodic nature of hydrocele surgical campaigns often partner-supported and periodically intensified when resources permit [30] compared to the continuous and community-based nature of lymphoedema care, which requires sustained availability of hygiene supplies, regular follow-up, and ongoing health worker engagement [31].

The particularly low coverage of lymphoedema management is concerning given the chronic, progressive nature of the condition and its strong association with recurrent acute dermatolymphangioadenitis (ADLA), disability, and stigma. Evidence from multiple countries shows that low-cost hygiene-based interventions can significantly reduce acute episodes and improve quality of life when consistently implemented [32,33]. Scaling up these interventions must therefore be prioritised within the DRC’s national LF strategy.

### Determinants of morbidity management coverage

Multivariable analyses identified clear determinants of access to morbidity management services. External partner support was strongly associated with hydrocele surgery coverage, highlighting the extent to which LF morbidity services rely on donor-funded activities. While external support has facilitated critical gains, its episodic nature raises concerns regarding long-term sustainability [34].

Across conditions, the presence of trained personnel and consistent availability of essential supplies emerged as central determinants of improved coverage. These findings align with evidence from other LF-endemic countries, where integration of MMDP services into primary healthcare systems has strengthened continuity, sustainability, and equity [35].

Conversely, provinces in the post–Transmission Assessment Survey (post-TAS) phase exhibited significantly lower morbidity management coverage. This suggests that, when transmission metrics improve, programmatic attention to MMDP often declines—a paradox identified in other countries [36]. This decline poses a direct risk to achieving WHO validation of LF elimination, which requires clear evidence of access to morbidity management services across all endemic areas.

### Strengths and limitations

This study has several important strengths. It draws on nationally representative programmatic data collected over seven years, enabling a comprehensive assessment of morbidity burden and service coverage across all endemic provinces. The combination of descriptive burden analysis, care cascade evaluation, and multivariable regression offers a robust understanding of both programmatic performance and system-level determinants of access to care. Hydrocele eligibility was confirmed using transillumination, improving diagnostic specificity and reducing misclassification. The use of community-based registries collected by community drug distributors during door-to-door MDA also provides a more accurate reflection of morbidity burden compared with facility-based reporting systems.

However, several limitations should be acknowledged. Data were aggregated and derived from routine programme reports, which may be subject to under-reporting and variability in data quality across provinces. Analyses conducted at the provincial level reduce geographic granularity and may mask substantial intra-provincial differences in access to morbidity management, raising the potential for ecological bias. Moreover, the absence of patient-level clinical information prevents assessment of disease severity, treatment outcomes, or the long-term impact of morbidity management. Although provincial-level analyses were necessary for comparability, morbidity management activities are implemented at the health zone level, meaning that sub-provincial heterogeneity may not be fully captured.

In addition, morbidity cases identified through CDD-based surveillance may underestimate the true burden compared with formal clinical examination, as previously reported in LF morbidity mapping studies [37,38].

### Implications for policy and practice

Despite these limitations, our findings have immediate and actionable implications for LF elimination efforts in the DRC and similar high-burden settings. Strengthening MMDP services, particularly lymphoedema management, must be prioritised alongside ongoing transmission monitoring. Key actions should include:

- integrating morbidity management into routine primary healthcare
- scaling up training of frontline health workers
- ensuring continuous availability of hygiene and wound-care supplies
- strengthening community-based detection and follow-up
- expanding access to hydrocele surgery, including through routine services
- securing predictable domestic and partner financing mechanisms

The findings also underscore the need for sustained support in post-TAS provinces to prevent programmatic decline at the very moment when community-based morbidity care becomes most essential.

Particular attention should be given to strengthening the pre-service and in-service training of physicians, nurses, and community health workers involved in LF morbidity management. Sustained capacity building, regular supportive supervision, and integration of the essential package of care into routine primary healthcare services are critical to improve continuity, quality, and equity of access, particularly in hard-to-reach and post-TAS settings.

## Conclusions

This national retrospective analysis demonstrates that lymphatic filariasis–related morbidities remain a substantial, persistent, and unevenly addressed public health challenge in the Democratic Republic of the Congo. Despite measurable progress in interrupting transmission, access to morbidity management services for both hydrocele and lymphoedema remains critically low, characterized by pronounced geographic disparities and significant attrition at every stage of the care cascade.

Strengthening and institutionalizing morbidity management and disability prevention within routine health services is essential to bridge these gaps. Priority actions include expanding community-based lymphoedema care, ensuring continuous availability of essential hygiene and wound-care supplies, scaling up access to safe hydrocele surgery, and investing in sustained training and supportive supervision of frontline health workers. Equally important is the establishment of stable financing mechanisms and reliable supply chains to reduce dependence on external partners and improve long-term sustainability.

Addressing these persistent weaknesses is vital for improving patient quality of life and reducing the burden of preventable disability. Critically, these improvements are also required for the Democratic Republic of the Congo to meet World Health Organization criteria for validation of lymphatic filariasis elimination as a public health problem. Without significant and sustained investment in morbidity management, progress toward elimination risks stalling despite advances in transmission control.

## Supporting information

S1 Table Provincial-level dataset used for regression analyses. This file contains the aggregated provincial dataset used to construct the multivariable regression models for hydrocele surgery coverage and lymphoedema morbidity management coverage. Variables include hydrocele and lymphoedema caseloads, number of surgeries performed, care coverage, partner support, trained personnel, accessibility indicators, and post–Transmission Assessment Survey status.
(DOCX)

S2 Table Summary of lymphatic filariasis morbidity management activities by year (2018–2024). This file presents the annual national totals of identified hydrocele and lymphoedema cases, eligibility assessments, hydrocele surgeries performed, and lymphoedema care delivered across all endemic provinces. It summarises temporal trends in morbidity management activities over the seven-year period.
(DOCX)
