# Supplementary material for: Geographical disparities and programmatic determinants of hydrocele surgery and lymphoedema management coverage for lymphatic filariasis in the Democratic Republic of the Congo, 2018–2024: A national analysis of routine programme data
Source: PLoS Negl Trop Dis. 2026 Jun 2;20(6):e0014406. doi: 10.1371/journal.pntd.0014406 (PMC13249136; doi:10.1371/journal.pntd.0014406)
Supplement: S1 Table — This file contains the aggregated provincial dataset used to construct the multivariable regression models for hydrocele surgery coverage and lymphoedema morbidity management coverage. Variables include hydrocele and lymphoedema caseloads, number of surgeries performed, care coverage, partner support, trained personnel, accessibility indicators, and post–Transmission Assessment Survey status. (DOCX) [file pntd.0014406.s001.docx]

**S1 Table. Provincial-level dataset used for regression analyses (2018–2024)**

*This table contains the aggregated provincial dataset used to construct the multivariable regression models assessing determinants of hydrocele surgery coverage and lymphoedema morbidity management coverage in the Democratic Republic of the Congo. All values correspond to the provincial totals and programmatic indicators presented in Tables 2 and 3 of the manuscript.*

| **Province / Coordination** | **Hydrocele cases** | **Hydrocele surgeries** | **Hydrocele coverage (%)** | **Lymphoedema cases** | **Lymphoedema care** | **Lymphoedema coverage (%)** | **Partner support (yes/no)** | **Trained surgeons (n)** | **OR nurses (n)** | **Caseload category** | **Post-TAS (yes/no)** | **Hard-to-reach (yes/no)** |
| --- | --- | --- | --- | --- | --- | --- | --- | --- | --- | --- | --- | --- |
| Kongo Central | 77 | 49 | 63.6 | 118 | 21 | 17.8 | Yes | 2 | 5 | Low | Yes | No |
| Tshopo | 758 | 275 | 36.3 | 1,203 | 138 | 11.5 | Yes | 3 | 10 | High | No | Yes |
| Nord Ubangi | 732 | 412 | 56.3 | 684 | 97 | 14.2 | Yes | 2 | 8 | Medium | No | Yes |
| Kwilu | 591 | 232 | 39.3 | 903 | 146 | 16.2 | Yes | 2 | 6 | Medium | Yes | Yes |
| Kasaï Central | 834 | 150 | 18.0 | 742 | 82 | 11.0 | No | 1 | 3 | High | Yes | Yes |
| Kasaï | 308 | 166 | 53.9 | 476 | 84 | 17.6 | Yes | 2 | 4 | Medium | No | Yes |
| Maniema | 284 | 112 | 39.4 | 355 | 63 | 17.7 | Yes | 1 | 4 | Low | Yes | Yes |
| Haut Katanga | 68 | 47 | 69.1 | 126 | 27 | 21.4 | Yes | 2 | 5 | Low | No | No |
| Ituri | 348 | 0 | 0.0 | 703 | 0 | 0.0 | No | 0 | 0 | High | Yes | Yes |
| Other provinces | 4,471 | 570 | 12.7 | 1,000 | 219 | 21.9 | Mixed | Variable | Variable | Mixed | Mixed | Mixed |

**Notes**

Coverage values correspond to programmatic data validated in Table 2 of the manuscript.

- Determinant variables (partner support, number of surgeons, OR nurses, post-TAS status, caseload category, accessibility) reflect the characteristics used in the regression models presented in Table 3.
- Caseload category (Low / Medium / High) was derived from total morbidity reported per province.
- “Other provinces” aggregates remaining endemic provinces to ensure consistency with national totals.
