# Supplementary material for: Geographical disparities and programmatic determinants of hydrocele surgery and lymphoedema management coverage for lymphatic filariasis in the Democratic Republic of the Congo, 2018–2024: A national analysis of routine programme data
Source: PLoS Negl Trop Dis. 2026 Jun 2;20(6):e0014406. doi: 10.1371/journal.pntd.0014406 (PMC13249136; doi:10.1371/journal.pntd.0014406)
Supplement: S2 Table — This file presents the annual national totals of identified hydrocele and lymphoedema cases, eligibility assessments, hydrocele surgeries performed, and lymphoedema care delivered across all endemic provinces. It summarises temporal trends in morbidity management activities over the seven-year period. (DOCX) [file pntd.0014406.s002.docx]

**S2 Table. Summary of lymphatic filariasis morbidity management activities by year, Democratic Republic of the Congo (2018–2024)**

*This table summarises annual lymphatic filariasis morbidity management activities nationwide, including case identification, eligibility assessment, and access to hydrocele surgery and lymphoedema care. Data reflect national programmatic reports aggregated from community drug distributor surveys and provincial NTD coordination offices.*

| **Year** | **Hydrocele cases identified** | **Hydrocele surgeries performed** | **Hydrocele coverage (%)** | **Lymphoedema cases identified** | **Lymphoedema cases receiving care** | **Lymphoedema coverage (%)** | **Notes** |
| --- | --- | --- | --- | --- | --- | --- | --- |
| **2018** | 1,120 | 240 | 21.4% | 690 | 110 | 15.9% | Start of intensified morbidity reporting via CDDs |
| **2019** | 1,305 | 310 | 23.8% | 720 | 118 | 16.4% | Provincial expansion of hydrocele campaigns |
| **2020** | 1,470 | 350 | 23.8% | 760 | 122 | 16.1% | COVID-19 disruptions; reduced surgical missions |
| **2021** | 1,650 | 420 | 25.5% | 810 | 135 | 16.7% | Resumption of surgical missions post-COVID |
| **2022** | 1,820 | 390 | 21.4% | 855 | 140 | 16.4% | MMDP budget constraints in several provinces |
| **2023** | 1,950 | 200 | 10.3% | 905 | 150 | 16.6% | Reduced partner support; transition to post-TAS |
| **2024** | 1,156 | 203 | 17.6% | 570 | 102 | 17.9% | Reallocation of resources to surveillance |

**Notes**

- Data reflect aggregated national reports; annual variations represent differences in partner support, training cycles, COVID-19 disruptions, logistical constraints, and timing of surgical missions.
- Hydrocele surgeries include routine and campaign-based interventions.
- Lymphoedema care includes hygiene-based self-care, ADLA management, wound care, and community follow-up.
- Coverage percentages are calculated using annual totals of cases identified.
- The decline in 2023 reflects programmatic reorientation toward post-TAS surveillance and reduced external funding.
